# Noncoding RNA actions through IGFs and IGF binding proteins in cancer

**DOI:** 10.1038/s41388-022-02353-3

**Published:** 2022-05-21

**Authors:** Aidan Kerr, Robert C. Baxter

**Affiliations:** University of Sydney, Faculty of Medicine and Health, Kolling Institute, Royal North Shore Hospital, St Leonards, Sydney, NSW 2065 Australia

**Keywords:** Oncogenes, Growth factor signalling

## Abstract

The insulin-like growth factors (IGFs) and their regulatory proteins—IGF receptors and binding proteins—are strongly implicated in cancer progression and modulate cell survival and proliferation, migration, angiogenesis and metastasis. By regulating the bioavailability of the type-1 IGF receptor (IGF1R) ligands, IGF-1 and IGF-2, the IGF binding proteins (IGFBP-1 to -6) play essential roles in cancer progression. IGFBPs also influence cell communications through pathways that are independent of IGF1R activation. Noncoding RNAs (ncRNAs), which encompass a variety of RNA types including microRNAs (miRNAs) and long-noncoding RNAs (lncRNAs), have roles in multiple oncogenic pathways, but their many points of intersection with IGF axis functions remain to be fully explored. This review examines the functional interactions of miRNAs and lncRNAs with IGFs and their binding proteins in cancer, and reveals how the IGF axis may mediate ncRNA actions that promote or suppress cancer. A better understanding of the links between ncRNA and IGF pathways may suggest new avenues for prognosis and therapeutic intervention in cancer. Further, by exploring examples of intersecting ncRNA-IGF pathways in non-cancer conditions, it is proposed that new opportunities for future discovery in cancer control may be generated.

## Introduction

### Long non-coding RNAs

Although 70% of the genome is transcribed into RNA, only 2% is translated into proteins, the remaining transcripts being either short or long noncoding (nc) RNAs [1]. Long ncRNAs (lncRNAs) are defined as those exceeding 200 nucleotides. The NONCODE v.6 database (www.noncode.org) recognizes over 170,000 human lncRNA transcripts from almost 100,000 genes. While their biological significance and mechanisms of action are still being elucidated, they have been implicated in a wide range of biological functions including development and disease progression. Most lncRNAs are transcribed by RNA polymerase II, a similarity shared with mRNA [2].

Depending on their structure lncRNAs can be subdivided into several groups which include long intergenic ncRNA (lincRNA), antisense lncRNA, and circular RNA. Post-transcriptionally lncRNAs regulate splicing, protein translation and stability and mRNA decay. The highly heterogeneous nature of lncRNAs has led them to be implicated in numerous different human diseases including cancer, cardiovascular, and neurodegenerative diseases [3], as reviewed extensively in recent years [4–7].

Most lncRNAs are thought to be *cis*-acting, i.e. they function near their site of transcription. Their roles include chromatin modification and transcriptional regulation of nearby genes. In contrast, the actions of *trans*-acting lncRNAs may be far from their transcription site, for example in the cytoplasm [8]. *Trans*-acting lncRNAs may regulate gene expression by interacting directly with DNA or chromatin complexes, serve as a scaffold to assist nuclear transcription and RNA processing, or control intracellular trafficking, cell-cell signaling and protein regulation through interaction with different macromolecules including proteins and RNA [2]. *Trans*-acting lncRNAs that “sponge” or sequester microRNA are examples of competing endogenous RNA (ceRNA).

### MicroRNAs

MicroRNAs (miRNAs) are a subtype of short ncRNA involved in the regulation of gene expression. Since the discovery of the first miRNA, Lin-4, in 1993, miRNAs have been implicated in an expanding range of biological functions. They are, on average, 22 nucleotides long with most miRNA precursors transcribed by RNA polymerase II or III before undergoing two cleavage events—nuclear and cytoplasmic—to form mature miRNA [9]. The two strands of a miRNA duplex may be designated with the suffix -5p (originating from the 5' side of the pre-miRNA), or -3p (from the 3' side) [10].

The human genome is reported to encode over 2650 mature miRNAs [11], which typically decrease gene expression post-transcriptionally, acting in a tumor-suppressive manner if they downregulate oncogenes. Many miRNAs achieve this by interacting with the 3'-untranslated region of target mRNAs resulting in mRNA destabilization, or they may act during translation to suppress initiation, cause premature dissociation from the ribosome, or influence degradation of the nascent polypeptide [12]. However, miRNA interaction with the 5'-untranslated region of mRNA, resulting in enhancement of translation, has also been described [13].

MiRNAs are essential in normal development and other biological processes, yet abnormal expression of miRNAs is implicated in many diseases [14]. MiRNAs are normally intracellular but are also found extracellularly as circulating miRNAs which exist in all body fluids, suggesting they may have potential to act as disease biomarkers. MiRNAs are hypothesized to target at least 60% of genes in humans and because they are evolutionarily conserved, they are assumed to be important for biological functioning [15].

### The IGF-IGFR-IGFBP axis

The insulin-like growth factor (IGF) axis encompasses the growth factors IGF-1 and IGF-2, the type 1 and 2 IGF receptors (IGF1R and IGF2R), and a group of six binding proteins (IGFBP-1 to -6) with high affinity for IGF-1 and IGF-2. These proteins initiate, or occupy a critical position in, a wide expanse of signaling networks. The fundamentals of IGF structure and physiology are well understood [16]. IGF-1 and IGF-2 bind with high affinity to the transmembrane tyrosine kinase receptor IGF1R, and with lower affinity to the structurally related insulin receptor (InsR), the exception being high-affinity binding of IGF-2 to the InsR isoform A. Conversely, insulin has much greater affinity for InsR than for IGF1R [16].

In the circulation and tissues the vast majority of IGF-1 and IGF-2 are bound to IGFBPs—predominantly IGFBP-3 in the adult circulation. Of the six IGFBPs, only IGF-occupied IGFBP-3 and -5 interact with a glycoprotein termed the acid-labile subunit to form ternary complexes which are the major carriers of circulating IGFs, increasing IGF half-life, retarding their extravascular transit to the tissues, and inhibiting their binding to both IGF1R and InsR at the tissue level [17]. The IGFBPs have a central role in modulating the oncogenic actions of IGFs mediated by IGF1R signaling, but they also have well-documented functions that occur independently of IGF- and IGF1R-dependent pathways [17]. Because their affinity for IGF-1 and IGF-2 is typically greater than the affinity of IGFs for IGF1R, IGFBP-bound IGFs may be restricted from activating IGF1R.

Following IGF1R autophosphorylation in response to IGF binding, its kinase activity initiates a cascade of downstream signaling events. Among the networks responsive to IGF1R activation, primary pathways include the PI3K/AKT/mTOR and Ras/Raf/MEF/ERK signaling cascades [18]. Activation of these pathways stimulates cellular proliferation and survival signaling, inhibiting apoptotic cell death and potentially promoting malignancy and carcinogenesis.

The IGF-2 receptor (IGF2R), also known as the cation-independent mannose 6-phosphate receptor, has an entirely different structure from the IGF1R, with no tyrosine kinase domain. In contrast to IGF1R, IGF2R negatively regulates proliferation by sequestering IGF-2 which is then unavailable to bind to IGF1R [19]. Therefore, IGF2R may act as a tumor suppressor by preventing IGF1R activation. Conversely, loss of *IGF2R* in malignant cells results in increased IGF-2 binding to IGF1R, increasing proliferation [19].

In cancers with upregulation of IGF1R and circulating IGF-1 levels, tumor growth may be stimulated by IGF1R activation. The potentially oncogenic action of IGF1R is readily demonstrated using IGF1R inhibitors in cell and animal models. Despite this, repeated clinical trials of IGF1R inhibitors, both monoclonal antibodies and small-molecule tyrosine kinase inhibitors, have failed to show clinical efficacy, either alone or in combinations, against several cancer types [20, 21]. In recent years numerous studies have demonstrated regulation of IGF1R by lncRNAs and miRNAs, suggesting that alternative targeting possibilities may be discovered by investigating these interactions. This topic has generated considerable interest in recent years and has been extensively reviewed by others [22–24].

## Noncoding RNA regulation of IGF-1 and IGF-2

Noncoding RNAs have the potential to influence the IGF-IGFR-IGFBP axis at every level, modulating the expression of the IGF ligands, receptors, and binding proteins. This section reviews selected studies that illustrate the regulation of the IGF1R ligands IGF-1 and IGF-2 by ncRNAs.

### Let-7

**Fig. 1:**
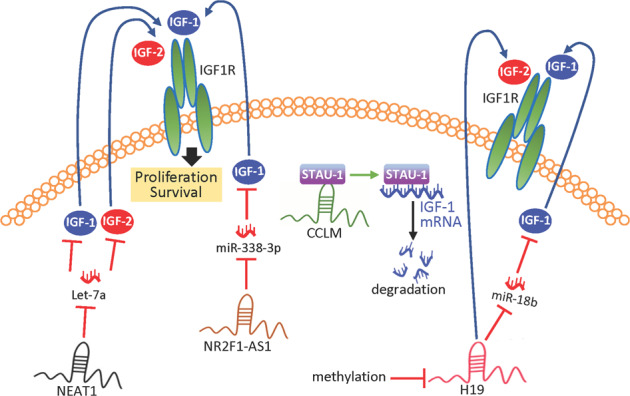
Proposed lncRNA regulation of IGF-1 and IGF-2. LncRNA NEAT1 increases oncogenic IGF1R signaling by competing against miRNA Let-7a, which targets IGF-2 [25]. Let-7 also potentially downregulates IGF-1 [26]. LncRNA NR2F1-AS1 can also lead to IGF1R activation by sponging miR-338-3p, which downegulates IGF-1 [27]. LncRNA CCLM interacts with the RNA-binding protein STAU-1, promoting its degradation of IGF-1 mRNA [28]. *H19* suppression by promoter methylation leads to an increase in IGF-2 expression [31]. H19 also increases IGF-1 by downregulating miR-18b, which targets IGF-1 [32].

A prime example is the miRNA let-7 family members, which have been extensively studied as potential tumor suppressors, targeting a variety of cell functions including cell cycle, differentiation and stemness, and immune function. Not always recognized is the significant role of let-7 members as regulators of IGF function. In NSCLC the lncRNA NEAT1 competes against let-7a, increasing cellular proliferation and metastasis. Let-7a targets IGF-2, which is oncogenic in NSCLC tissue [25]; increased expression of NEAT1 downregulates let-7a, removing the miRNA block on IGF-2 expression, and resulting in increased NSCLC cell proliferation, migration and invasion [25]. Similarly, in epithelial ovarian cancer, let-7 also downregulates IGF-1, possibly contributing to a less aggressive phenotype [26] (Fig. 1).

Among other ncRNAs that regulate IGF expression, lncRNA NR2F1-AS1 appears oncogenic in several cancers including thyroid, endometrial, hepatocellular and breast. In breast cancer models NR2F1-AS1 increased IGF-1 expression and tumor formation. NR2F1-AS1 was shown to act as a ceRNA by sponging miR-338-3p, preventing it from downregulating IGF-1 [27]; the resulting increase in IGF-1 activated IGF1R in endothelial cells, increasing ERK signaling and breast cancer angiogenesis [27]. In a contrasting mechanism, lncRNA lncCCLM is a potential tumor suppressor in cervical cancer, interacting in the cytoplasm with the RNA-binding protein STAU1 to promote its binding and degradation of IGF-1 mRNA (Fig. 1). The resulting loss of IGF-1 results in decreased lymphatic metastasis [28].

### H19

The gene encoding IGF-2 is closely co-located with that for lncRNA H19 on human chromosome 11p15. These genes are reciprocally imprinted, with expression of the paternal *IGF2* allele and the maternal *H19* allele. H19 plays a role in numerous tumorigenic pathways and is one of the most extensively studied lncRNAs [29]. One proposed role of H19 is to regulate IGF-2 expression; specifically, mice inheriting a maternal targeted deletion of *H19* show overexpression of IGF-2 [30]. Reciprocal regulation of IGF-2 and H19 expression is seen in adrenocortical carcinoma, where H19 suppression by promoter methylation is associated with increased IGF-2 expression and cell proliferation [31]. Contrasting with its putative tumor suppressor role through IGF-2, H19 may also have an oncogenic role through IGF-1 regulation. In cisplatin-resistant melanoma cells H19 was shown to interact with miR-18b, downregulating its expression. Since IGF-1 is a target of miR-18b, miR-18b suppression by H19 upregulated IGF-1 to increase chemotherapy resistance; conversely, H19 silencing decreased cell survival [32] (Fig. 1).

## Regulation of IGFBPs by ncRNAs

As regulators of IGF signaling through IGF1R, IGFBPs were originally regarded as having a predominantly tumor suppressive role. Evidence for this IGF1R-dependent activity of IGFBPs is complemented by many studies showing antiproliferative and pro-apoptotic effects of IGFBPs acting independently of the modulation of IGF1R signaling. Contrasting with these growth-inhibitory actions of IGFBPs, there is also clear evidence of growth promotion by IGFBPs in both cancer and non-cancer cells, as well as extensive clinical data linking high IGFBP expression in a variety of cancers to a more aggressive phenotype and poor patient survival [17].

IGFBP-3 is the most abundant IGFBP in the adult human circulation, and provides many examples of the disparate growth-inhibitory and stimulatory actions of IGFBPs. Its dysregulation is associated with several cancers and its measurement in the circulation or in tumor tissues may also have prognostic utility [17]. It appears to act as a tumor suppressor in some cancers including hepatoma [33] and non-small cell lung cancer [34], where its downregulation in tumor tissue is associated with a worse prognosis. In contrast, in some other cancers including prostate cancer [35] and clear cell renal cell carcinoma [36] increased tumor IGFBP-3 expression may be associated with poor patient outcomes. Notably, these findings are not universal, and there may be differences between the prognostic significance of circulating compared to tissue IGFBP-3 levels [17]. For IGFBP-3 and the other five IGFBPs, the ambiguity of some clinical findings reflects the diverse actions observed in cell biology studies, emphasizing how incomplete our understanding of these important regulatory proteins remains. IGFBP interactions with ncRNAs add a further layer of complexity to their regulation and actions.

### IGFBP-1

IGFBP-1, a secreted phosphoprotein, is generally highly phosphorylated and inhibitory to IGF action; however a hypophosphorylated form of IGFBP-1 can potentiate IGF-1-mediated cell proliferation [17], and IGFBP-1 is known from non-cancer studies to promote migration through integrin signaling [37]. In lung cancer cells, miR-155-5p was found to downregulate the transcription factor FOXO3a, leading in turn to downregulation of IGFBP-1. Since IGFBP-1 has a tumor suppressor role in lung cancer, its suppression results in enhanced cancer proliferation [38]. In gastric cancer cells IGFBP-1 has been reported as a target of miR-519a, but paradoxically when miR-519a is overexpressed (and IGFBP-1 downregulated), cell migration and invasion are inhibited, suggesting a possible tumor-promoting role for IGFBP-1 in this cancer [39]. In gastric cancer patient tissue miR-519a expression is low compared to control tissue, its low expression being prognostic for poor survival [39]. A potential tumor-suppressor role for miR-519a has also been described in other cancers, but further work is needed to establish whether IGFBP-1 downregulation has a broader role in its actions.

### IGFBP-2

IGFBP-2 has been characterized as exerting a unique influence on cancer metastasis, mediating the metastatic effect of miR-126 downregulation which has been observed in many cancers. IGFBP-2 was shown to be specifically involved in endothelial recruitment, angiogenesis, and metastatic colonization, an effect that required the presence of both IGF-1 and IGF1R [40]. The data suggest a potentiating effect of IGFBP-2 on IGF-1 signaling through IGF1R in endothelial cells. MiR-126 suppressed metastatic endothelial recruitment and metastatic colonization through targeting *IGFBP2* in addition to *PITPNC1* and *MERTK* genes [40].

In glioma cells IGFBP-2 is well known to act as an oncogene and its increased expression is associated with poor prognosis of GBM [41]. This appears to be driven in part by downregulation of miR-491, which is decreased in GBM tissue compared to healthy brain. MiR-491-3p was shown to negatively regulate the expression of both IGFBP-2 and the cell cycle effector CDK6, whereas the other miR-491 product, miR-491–5p targets EGFR and CDK6, leading to decreased cellular proliferation, invasion, and stem cell propagation [42]. Other miRNAs described as targeting IGFBP-2 in glioma cell models, decreasing cell migration, invasion and survival, include miR-204-3p [43] and miR-302b [44]. MiR-302b targets both IGFBP-2 and the transcription factor, nuclear factor IA (NFIA) [44], high expression of which is associated with poor survival of patients with GBM. Since NFIA transcriptionally upregulates *IGFBP2*, miR-302b suppression enhances IGFBP-2-dependent oncogenesis both directly, and indirectly through NFIA [44].

**Fig. 2:**
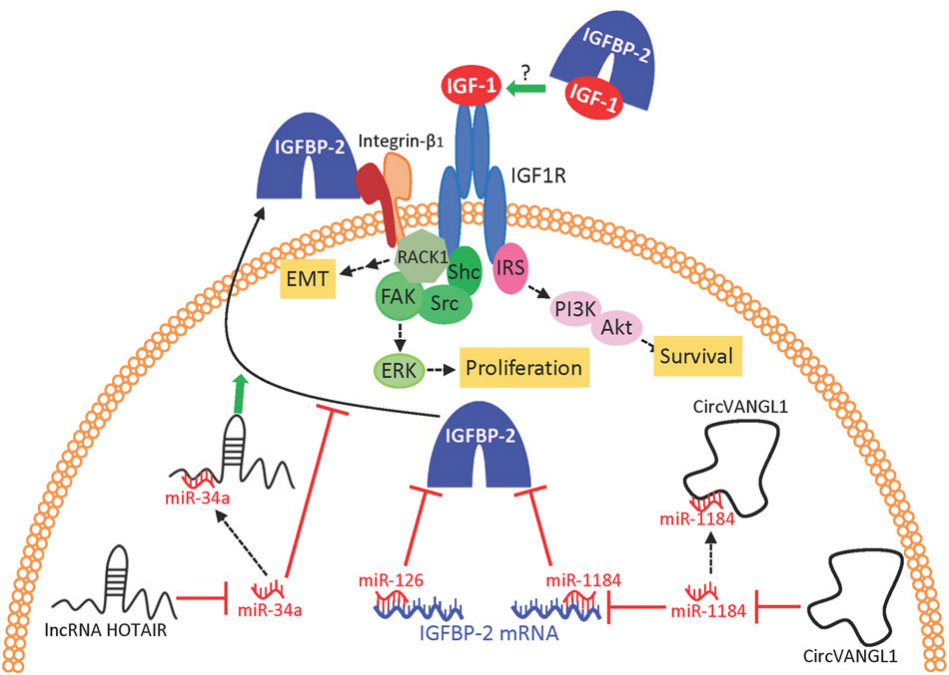
Potential oncogenic ncRNA actions through IGFBP-2. MiR-1184 [45] and miR126 [40] can bind to 3'-UTR sites of IGFBP-2 mRNA to repress its translation. CircVANGL1, which sponges miR-1184, increases IGFBP-2 expression [45]. HOTAIR also increases IGFBP-2, possibly mediated by sponging miR-34a [48, 49]. MiR-34a suppression promotes IGFBP-2 secretion by RAB6A^+^ vesicles [50]. IGFBP-2 stimulates oncogenic signaling through IGF1R [40], but whether this involves IGF binding by IGFBP-2 is unclear. A potential mechanism involves interaction with integrin-β1, which forms a signaling complex with IGF1R including the scaffold protein RACK1, leading to proliferative ERK activation and other oncogenic signaling [47].

Circular RNAs (circRNAs) are lncRNAs with a closed loop structure, lacking 5' and 3' polarity. Many circRNAs act by negatively regulating miRNA expression, preventing them from binding to RNA-binding proteins or preventing translation of proteins. CircVANGL1 is a circRNA upregulated in bladder cancer tissue which, when inhibited in vitro, decreases the oncogenic potential of bladder cancer cells [45]. It acts as a ceRNA or sponge for miR-1184, which interacts with the 3'UTR of the IGFBP-2 mRNA, inhibiting its expression and resulting in decreased cell migration, invasion and growth. Therefore, circVANGL1 is believed to act as a tumor promoter through IGFBP-2 upregulation [45] (Fig. 2).

Although the precise mechanism of IGFBP-2 oncogenesis in bladder cancer (or other cancers) is unclear, it has a defined role in stimulating angiogenesis and metastasis through IGF1R as described above [40], and its reported oncogenic signaling through integrin-β_1_ and ERK activation [46] is consistent with the known interaction between activated IGF1R and integrin-β_1_ with scaffolding by RACK1 and FAK, which promotes proliferative ERK signaling as well as EMT and increased invasiveness [47] (Fig. 2).

The lncRNA Hox antisense intergenic RNA (HOTAIR) has a role in metastasis in several cancers including renal cell carcinoma (RCC), in which elevated HOTAIR expression is prognostic for poor patient survival [48]. Stimulation of IGFBP-2 expression by HOTAIR appears to mediate this effect, since enhanced cell migration and tumor growth in vivo resulting from HOTAIR overexpression was reversed by IGFBP-2 silencing [48]. This proposal is supported by IGFBP-2 and HOTAIR co-expression in clinical RCC samples, and very poor survival data for patients with tumors that highly express both markers. In addition to its effect on IGFBP-2 expression, HOTAIR may independently increase IGFBP-2 secretion since it downregulates expression of miR-34a and acts as a miR-34a-5p sponge [49]. MiR-34a downregulation is reported to increase vesicle-mediated extracellular release of IGFBP-2 [50]. Thus independent studies suggest that HOTAIR may exert an oncogenic role through IGFBP-2 by stimulating both its expression and secretion (Fig. 2).

### IGFBP-3

IGFBP-3 provides the main circulating reservoir for IGF-1 and IGF-2 in adults. As described earlier, at the cellular level IGFBP-3 can act either as an oncogene or a tumor suppressor in different cancers. Its inhibitory actions may result from blockade of IGF1R signaling by binding IGF-1 and IGF-2, or through mechanisms independent of IGF1R, while its stimulatory actions are in some cases mediated through activation of sphingosine kinase 1 [17].

In osteosarcoma cells IGFBP-3 expression has been shown to be stimulated by miR-384, resulting in decreased cell proliferation, EMT, and invasion [51]. Expression of both miR-384 and IGFBP-3 was lower in osteosarcoma tissue than healthy tissue, and lowest in high-stage cancers. Therefore, in osteosarcoma it is proposed that miR-384 acts as a tumor suppressor through upregulation of IGFBP-3, with low expression of both associated with poor patient outcomes [51]. In contrast to this study, IGFBP-3 downregulation by miRs has been more commonly observed. High IGFBP-3 expression in glioblastoma (GBM) tissue has been associated with either better [52] or worse [53] patient outcomes. In a study where IGFBP-3 appeared to act as a tumor suppressor, miR-21, which is oncogenic and highly expressed in GBM tissue, was shown to downregulate IGFBP-3 [52]. The inhibition of xenograft GBM tumor growth by miR-21 silencing was reversed by IGFBP-3 knockdown, suggesting that miR-21 oncogenesis is mediated by IGFBP-3 suppression. However, the disparate clinical findings on the role of IGFBP-3 in GBM indicate that further mechanistic and clinical studies are warranted.

In ovarian cancer miR-19a-3p expression is elevated compared to healthy tissue and appears to have an oncogenic role [54]. MiR-19a-3p targets the expression of IGFBP-3, which is tumor-suppressive in this cancer; thus miR-19a-3p increases the growth and invasiveness of ovarian cancer cells in vitro, and the growth of xenograft tumors in vivo [54]. This suggests that IGFBP-3 downregulation may mediate the oncogenic action of high miR-19a-3p expression in ovarian cancer [54]. Similarly in p53-mutant NSCLC cell lines, miR-125b was found to downregulate IGFBP-3 leading to a more invasive phenotype, associated with increased PI3K/AKT signaling [55]. Since high miR-125b expression in NSCLC tumors is strongly associated with a decrease in overall and recurrence-free patient survival, and is significantly correlated with low IGFBP-3 expression, this study points to miR-125b-dependent IGFBP-3 downregulation as a contributory factor in NSCLC [55].

In a similar manner, in colorectal cancer cells overexpression of miR-197 caused increased migration and invasion concomitant with downregulation of IGFBP-3 expression. The miR-197 levels in colorectal cancer tissues were inversely correlated with expression of IGFBP-3 and were associated with a more aggressive phenotype [56]. MiR-197 is also highly expressed and related to poor prognosis in gallbladder cancer [57]. In cell models downregulation of miR-197 decreased proliferative signaling and increased apoptotic markers. miR-197 was shown to directly target IGFBP-3, leading to a more aggressive phenotype. Therefore, as in colorectal cancer, IGFBP-3 may mediate the oncogenic effect of miR-197 [57]. A comparable role for IGFBP-3 downregulation in mediating miR-197-dependent oncogenesis has been reported in nephroblastoma [58]. In contrast to many studies in which IGFBP-3 is tumor-suppressive, IGFBP-3 seems to act as an oncogene in gastrointestinal stromal tumors (GIST), in which low expression of miR-186 is proposed to increase metastasis through the upregulation of IGFBP-3 and several other metastatic genes [59]. MiR-186 measurement is hypothesised to act as a predictive biomarker for clinical recurrence of GIST, but the precise role of IGFBP-3 needs further confirmation.

Another illustration of IGFBP-3 acting as an oncogene relates to the circRNA, circ-0046263, which is upregulated in nasopharyngeal carcinoma (NPC), with highest expression in metastatic disease [60]. In NPC cell lines, circ-0046263 silencing decreases proliferation, migration and invasion. Cytoplasmic circ-0046263 acts as a sponge for miR-133a-5p; miR-133a-5p in turn downregulates IGFBP-3 [60]. As a result, the level of IGFBP-3 was found to be significantly elevated in rapidly-growing NPC xenograft tumors that overexpress circ-0046263. These results are consistent with a direct role for circ-0046263, acting through miR-133a-5p, in promoting high IGFBP-3 expression and poor patient outcome in NPC tumors [60]. In the parallel example of osteosarcoma, high expression of circRNA circ-0000285 was found to sponge the miRNA miR-409-3p, which in turn led to upregulation of IGFBP-3 [61]. Circ-0000285 silencing decreased the oncogenic phenotype in vitro and inhibited xenograft tumor growth in mice, with a concomitant reduction in tumor IGFBP-3. This study was interpreted as indicating that in osteosarcoma, circ-0000285 is a ceRNA which, by sponging miR-409-3p, acts as a tumor promoter, upregulating oncogenic IGFBP-3 [61].

**Fig. 3:**
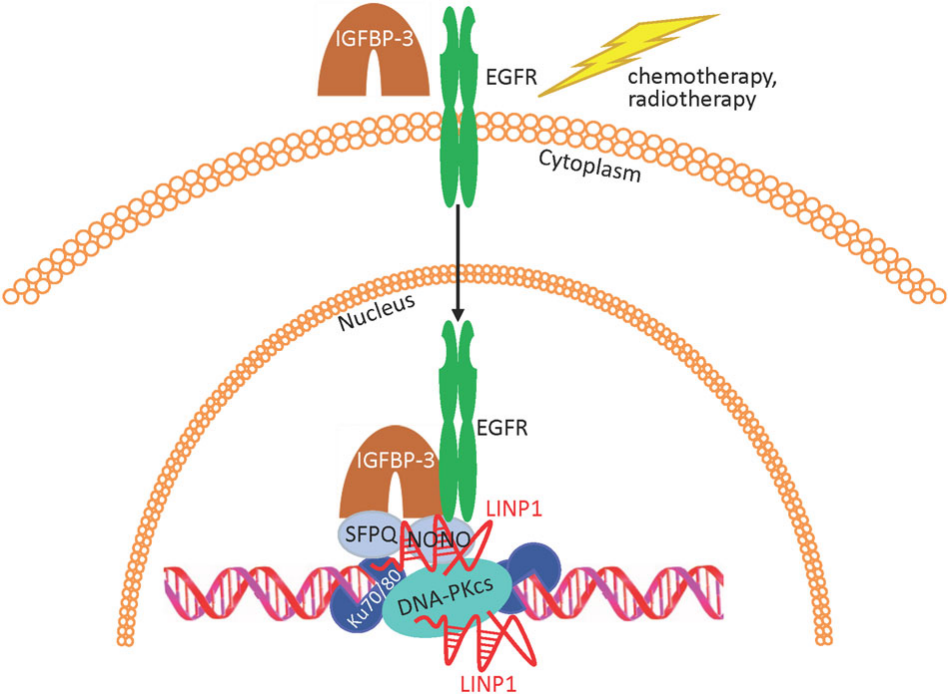
IGFBP-3, LINP1, and DNA damage repair. In cells exposed to chemotherapy, lncRNA LINP1 facilitates DNA double-strand-break repair by nonhomologous endjoining, acting as a scaffold between the repair complex proteins Ku80 and DNA-PKcs [62]. EGFR and IGFBP-3 form part of this complex, translocating to the nucleus and interacting with DNA-PKcs in response to chemotherapy [63]. RNA-binding proteins NONO and SFPQ are also found in the complex after chemotherapy, interacting with IGFBP-3 [64]. This interaction involves LINP1 and is prevented by LINP1 silencing [64]. LncRNA NEAT1 has also been reported as part of a similar nuclear complex involving DNA-PKcs, NONO, and SFPQ [65].

In triple-negative breast cancer (TNBC), LINP1 (lncRNA in nonhomologous end joining (NHEJ) pathway 1) acts in the NHEJ pathway of DNA damage repair, serving as a scaffold between DNA-PKcs and Ku80 in the multi-protein DNA repair complex [62]. In response to chemotherapy, IGFBP-3 and EGFR translocate to the nucleus and interact with DNA-PKcs in the NHEJ complex; both IGFBP-3 and phospho-EGFR are required for DNA-PKcs activation and DNA endjoining to occur [63]. The RNA-binding proteins, NONO and SFPQ, also interact with IGFBP-3 and DNA-PKcs in response to chemotherapy, and this interaction requires the presence of LINP1; when LINP1 was silenced, IGFBP-3 interaction with NONO and SFPQ was blocked, preventing DNA repair after chemotherapy [64] (Fig. 3). Although IGFBP-3 can act as a tumor suppressor, its expression in breast cancer is highest in basal-like TNBC and correlates with shorter recurrence-free patient survival [17]. Another lncRNA potentially involved in a similar nuclear complex with DNA-PKcs is NEAT1, which preferentially binds to NONO and SFPQ, and like LINP1 can act as a scaffold. NEAT1 has been reported as part of a similar ribonuclear complex involved in the DNA-mediated innate immune response, that also contains, among other proteins, DNA-PKcs, Ku70/Ku80, NONO, and SFPQ [65], similar to the NHEJ complex that includes IGFBP-3. This suggests that NEAT1 and LINP1 may have parallel or related functions in nuclear complexes involving DNA-PKcs, NONO, and SFPQ (Fig. 3).

### IGFBP-4

Lnc-IGFBP4-1 is a recently-described lncRNA located in the upstream region of the *IGFBP4* gene. Lnc-IGFBP4-1 expression is increased in lung cancer tissue compared to non-cancerous tissue, high expression being associated with high TNM stage and lymph node metastasis [66]. In preclinical models lnc-IGFBP4-1 promoted cell proliferation and migration and xenograft tumor growth, with a modest effect on cell metabolic activity. An inverse relationship was observed between the expression of lnc-IGFBP4-1 and IGFBP-4 both in lung cancer tissues and cell models [66]. Since IGFBP-4 acts predominantly by sequestering IGF-1, blocking its access to IGF1R, it is suggested that the tumorigenic actions of lnc-IGFBP4-1 are dependent on IGFBP-4 downregulation and IGF1R activation, but this remains to be definitively demonstrated. In a parallel study of bladder cancer, lnc-IGFBP4-1 was similarly highly expressed in cancer tissues and oncogenic in cell models [67]. Lnc-IGFBP4-1 was found to stimulate STAT3 activation, inhibition of which suppressed cell cycle and promoted apoptosis. Although phospho-IGF1R was not measured in this study, STAT3 phosphorylation is a recognized response to IGF1R activation [68]; thus it may be surmised that the pathway of IGFBP4-1 oncogenic activity is suppression of IGFBP-4, leading to enhanced IGF1R signaling and subsequent STAT3 activation.

### IGFBP-5

Like IGFBP-3, IGFBP-5 has been extensively documented as having both inhibitory and stimulatory effects on the oncogenic phenotype in different experimental systems [17]. In papillary thyroid carcinoma (PTC), the most common form of thyroid cancer, IGFBP-5 expression is targeted by miR-204-5p [69], in contrast with the alternate precursor product, miR-204-3p, which was noted earlier as targeting IGFBP-2 in glioma [43]. MiR-204-5p interaction with the 3'-UTR of IGFBP-5 mRNA leads to IGFBP-5 downregulation, increased apoptosis, and cell cycle arrest, implying an oncogenic role for IGFBP-5 in this cancer. Low miR-204-5p expression is observed in PTC tissue samples compared to adjacent non-cancer tissue, with a parallel increase in IGFBP-5 expression, suggesting that the tumor-suppressor role of miR-204-5p in PTC depends on IGFBP-5 regulation [69]. Independent evidence suggests that this effect of miR-204-5p is controlled by lncRNA UCA1 (Urothelial Cancer Associated 1), which is oncogenic in many cancers and highly expressed in PTC tissues and cell lines [70]. UCA1 silencing in cell culture stimulates miR-204 expression and downregulates IGFBP-5, inhibiting the oncogenic phenotype, and these effects are reversed by miR-204 inhibition. Although pre-clinical and clinical confirmation is required, these studies suggest that PTC may in part be driven by UCA1 acting as a ceRNA to suppress miR-204-5p and stimulate oncogenic IGFBP-5 [70].

A parallel function may be served by circPIP5K1A in ovarian cancer [71]. CircPIP5K1A upregulation was found in ovarian cancer tissue samples, associated with poor patient survival, and in cell lines associated with increased proliferation, migration, and invasion [71]. CircPIP5K1A acts as a ceRNA to downregulate miR-661, which in turn targets IGFBP-5 expression. By sponging miR-661, circPIP5K1A may upregulate IGFBP-5, contributing to the oncogenic phenotype in ovarian cancer [71]. Again, in vivo and clinical support for this hypothesis is required.

As noted above, IGFBP-5 does not always act as an oncogene. A bioinformatic approach has been used to identify genes that are differentially regulated in liver metastases of colorectal cancer (CRC) compared to primary CRC tissue [72]. These included miR885 (upregulated) and IGFBP-5 (downregulated). This reciprocal relationship was confirmed in vitro, where miR885 stimulated migration in CRC cell lines, concomitant with downregulation of IGFBP-5. This was taken to suggest that in CRC, IGFBP-5 may inhibit cell migration and, by implication, metastasis, providing groundwork for further investigation [72]. A further example of IGFBP-5 potentially mediating tumor suppression by a ncRNA comes from lncRNA DIRC3, low expression of which is a poor prognostic indicator in melanoma patients [73]. *DIRC5* was shown to be located in close genomic proximity to *IGFBP5*, with *DIRC5* depletion acting in *cis* to suppress *IGFBP5* expression, mediated by regulation of the transcription factor SOX10. The resulting IGFBP-5 downregulation enhanced anchorage-independent growth of melanoma cell lines, the proposed mechanism being enhanced IGF1R signaling, although this was not demonstrated [73].

### IGFBP-6

Uc.416+A is a member of a class of ultraconserved ncRNAs with 100% conservation among human, rat and mouse genomes. In gastric cancer (GC) samples Uc.416+A is highly expressed compared to non-cancer gastric mucosa, inversely with the expression of its putative target IGFBP-6 which is low in GC [74]. Uc.416+A is downregulated by binding miR-153, and the two RNAs show inverse regulation in GC cell lines. Since Uc.416+A stimulates cell proliferation in culture, it was proposed that low miR-153 expression in GC may contribute to cancer progression by upregulating Uc.416+A which, in turn, decreases IGFBP-6, allowing enhanced IGF-dependent activation of IGF1R [74]. It is possible that the effect of suppressed IGFBP-6 might be modified by changes in IGF1R or INSR signaling at the gene expression level. An independent study in breast cancer cells has shown that IGFBP-6 silencing by shRNA induced expression of genes encoding miR-100 and let-7a, which are known to target IGF-2, IGF1R and INSR [75]. IGF-2 is the preferred high-affinity ligand of IGFBP-6, and can activate signaling through both IGF1R and isoform A of INSR. However this study did not include supportive cell biology studies and understanding its functional implications requires further investigation.

The examples above illustrate that each member of the high-affinity IGFBP family can mediate the action of ncRNAs. Notably, IGFBP-2, IGFBP-3, and IGFBP-5 participate in a wide variety of ncRNA-regulated pathways, with both stimulatory and inhibitory effects on cancer cell functions. This complexity of regulation may offer new avenues for therapeutic intervention in a wide range of malignancies.

## Lessons learnt from other diseases

While knowledge surrounding the interactions of IGF-axis proteins with miRNAs and lncRNAs in cancer is still expanding, discoveries in other diseases provide valuable insights and may stimulate ideas for future research. The following examples have been selected to illustrate links between ncRNAs and IGFs or IGFBPs in non-cancer contexts which suggest testable hypotheses on the mechanisms of action of miRNAs in cancer.

### Actions through IGF-1 and IGF-2

#### SNHG1–miR-450b–IGF-1

In a human cardiomyocyte model, the lncRNA SNHG1 (small nucleolar RNA host gene 1) has been shown to be cardioprotective, inhibiting apoptosis and decreasing oxidative stress after hypoxia/reoxygenation [76]. SNHG1 acts by sponging miR-450b-5p, a miRNA that downregulates IGF-1; thus SNHG1 can reverse the decline in IGF-1 expression and pro-survival Akt signaling after hypoxia/reoxygenation [76]. The stimulatory effect of SNHG1 on IGF-1, though not yet described in cancer models, may be highly relevant since SNHG1 has been extensively reported to be upregulated in a variety of cancers including colorectal, lung, prostate and bone cancers, with effects on cell proliferation, migration and invasion [77]. This suggests that the proposed SNHG1–miR-450b-5p–IGF-1 pathway should be investigated for its role in oncogenesis.

#### TUG1–miR-148b–IGF-2

The lncRNA TUG1 (taurine up-regulated gene 1) is believed to act in cardiovascular disease by enhancing atherosclerosis in part through an increase in vascular smooth muscle cell (VSMC) proliferation and survival [78]. Using VSMCs stimulated with oxidized LDL as a model of atherosclerosis, it was shown that TUG1 silencing increased miR-148b, leading to a decline in IGF-2 which was found to be a target of miR-148b [78]. TUG1 is overexpressed and oncogenic in many, though not all, cancers [79], but its potential actions through IGF-2 upregulation have not been explored in a cancer context. This study suggests that a TUG1–miR-148b–IGF-2 pathway might have an oncogenic role in some cancers.

### Actions through IGFBP-2

#### MiR-130b

MiR-130b-5p and miR-130b-3p, the two strands of the miR-130b duplex, appear to have independent actions on IGF-axis proteins in non-cancer models, which may be relevant in some cancers. In a murine model of non-alcoholic fatty liver disease, downregulation of miR-130b-5p, which targets IGFBP-2, prevented lipid accumulation and insulin resistance by increasing IGFBP-2 and AKT activation [80]. Similarly, miR-130b-3p downregulation, as observed in lung fibrosis, can stimulate cell proliferation by increasing IGF-1 expression [81]. These actions of miR-130b, although not revealed in cancer models, may shed light on miR-130b actions in cancer. For example, in cervical cancer increased miR-130b-5p was shown to downregulate the transcription factor ELK1 which resulted in decreased stemness, proliferation and migration of cervical cancer cells [82]. Since ELK1 is downstream of IGF1R activation through ERK/MAPK, IGF-1 is likely to have an intermediary role in this mechanism. In contrast, in gastric cancer overexpression of miR-130b-5p led to increased proliferation, migration and invasion, reportedly through its ability to target RAS protein activator like 1 (RASAL1) [83]. MiR-130b is upregulated in tissue and serum samples of HCC patients, leading to the proposed measurement of circulating levels of miR-130b as a potential tumor biomarker to diagnose HCC [84], although caution is required when extrapolating this to other cancers. Interactions of miR-130b-5p have also been described in other cancers including pancreatic, colorectal, nasopharyngeal and ovarian cancer [85–88], but the role of IGF-axis proteins in mediating miR-130b-5p actions in these cancers has not yet been established.

#### MiR-873

Parallel to the observation with miR-130b, downregulation miR-873 in a rat model of gestational diabetes was reported to reduce insulin resistance and myocardial injury by upregulating IGFBP-2, allowing promotion of PI3K/AKT/mTOR signaling. IGFBP-2 was shown to be a direct target of miR-873 [89]. MiR-873 is well known to promote cancer progression and development in several different cancer types, although no link to the IGF-axis has yet been reported. In breast cancer tissue, miR-873 was downregulated compared to normal mammary tissue, resulting in increased PD-L1, a target of miR-873 [90]. This was positively associated with increased stemness and chemoresistance, putatively through activation of PI3K/AKT and ERK1/2 pathways [90]. IGFBP-2 also upregulates PD-L1, and facilitates nuclear EGFR accumulation [91]; thus it may be hypothesized that the targeting of IGFBP-2 by miR-873 is also implicated in both stemness and chemoresistance in breast cancer. In human colorectal cancer (CRC), miR-873 is downregulated and this increases ELK1 expression [92] as described above for miR-130b. Again, IGF1R signaling is likely to be involved, as an activator of ELK1. Similarly in CRC, miR-873-5p inhibits cell migration, invasion and EMT through its targeting of ZEB1 [93]. Since IGF1R signaling upregulates ZEB1 [94], this suggests another potential point of intersection between the IGF axis and miR-873 actions in CRC.

### Actions through IGFBP-3

#### MiR-19a-3p

In a rat model of cerebral ischemia/reperfusion injury miR-19a-3p was found to promote reperfusion-induced inflammation and apoptosis by downregulating IGFBP-3—implying a neuroprotective effect of IGFBP-3 [95]. The similar protective effect of a miR-19a-3p inhibitor, resulting in a reduced infarct volume, better neurological outcomes together with enhanced cell viability and decreased inflammation, was reversed by IGFBP-3 silencing, suggesting that IGFBP-3 is anti-apoptotic in this model [95]. Translated to a cancer context, targeting of oncogenic IGFBP-3 by miR-19a-3p might contribute to the observation in colorectal cancer that lncRNA LINC00342, which is highly expressed and acts as ceRNA to sponge miR-19a-3p, promotes CRC cell growth and invasion [96]. Similarly in bone metastatic prostate cancer, miR-19a-3p downregulation promotes an oncogenic phenotype [97]. However, there are also examples of miR-19a-3p itself acting as an oncogene, so if IGFBP-3 downregulation is involved in these effects, this would imply a tumor-suppressive role of IGFBP-3.

### Actions through IGFBP-5

#### MiR-193

Members of the miR-193 family (miR-193a, miR-193b) have been linked to cancer in many studies, either promoting or suppressing tumorigenesis [98]. A potential tumor-suppressive mechanism is suggested by a study in which circulating miR-193b was shown to be downregulated in gestational diabetes patients compared to healthy controls, associated with increased apoptosis and autophagy through an increase in IGFBP-5 [99]. Similarly, in rheumatoid fibroblast-like cells, in which miR-193a-3p also targets IGFBP-5, miR-193a-3p inhibition leads to IGFBP-5 induction and enhanced apoptosis [100]. Therefore it may be speculated that induction of IGFBP-5, which is pro-apoptotic in some cancers [17], may be an important function of miR-193 downregulation in tumor suppression.

#### MiR-24-3p

In degenerative nucleus pulposus cells from patients with intervertebral disc degeneration, miR-24-3p was shown to target the 3’-UTR of IGFBP-5 mRNA, downregulating IGFBP-5 and enhancing ERK activation [101]. Since IGF1R commonly signals through ERK, this is consistent with IGFBP-5 binding IGFs to block IGF1R activation. IGFBP-5 targeting by miR-24-3p could be relevant in a number of cancers in which miR-24-3p has an oncogenic role, including lung [102], breast [103], and bladder cancer [104]. MiR-24-3p may be regulated by lncRNA CASC2, which has been shown in thyroid cancer cells to sponge miR-24-3p, inhibiting cell viability and invasion in vitro, and xenograft tumor growth in vivo [105]. Again, this observation is consistent with IGFBP-5 being a miR-24-3p target in thyroid cancer.

There are many other examples in the non-cancer literature of miRNA effects that involve IGFs and/or IGFBPs. The cases cited above may provide a starting point for the generation of new hypotheses that could further elucidate the role of IGF-axis proteins in mediating the actions of noncoding RNAs in cancer.

## Prognostic and therapeutic opportunities

### Prognosis

A current limitation of many of the functional studies discussed in this review is that they are generally based only on cell biology experimentation, in some cases with pre-clinical confirmation in vivo. This provides an important basis for future clinical investigation, but few of these clinical studies have yet been undertaken. Many reports in the cited literature claim a potential prognostic role for ncRNAs, both miRNAs and lncRNAs, covering virtually every cancer type; however, beyond the demonstration of differential ncRNA expression between cancer and corresponding healthy tissue, strong clinical validation among the cited studies is often lacking.

Among the many putative ncRNA-IGF pathways discussed in this review, some are supported by patient survival analyses and tissue investigations. For example, the clinical relevance of a HOTAIR-IGFBP-2 pathway in renal cell carcinoma is demonstrated by Kaplan-Meier analyses showing decreased cancer-specific survival associated with high expression of both HOTAIR and IGFBP-2, with lowest survival when both are high; plus HOTAIR and IGFBP-2 colocalization in tissue sections, and a significant correlation between tissue HOTAIR and IGFBP-2 expression [48]. Supported by mechanistic cell biology studies, this provides a firm basis to further explore the prognostic value of the HOTAIR-IGFBP-2 axis in RCC. A previously-discussed study of miR-125b and its target IGFBP-3 in NSCLC similarly provides a sound rationale for further investigation as a prognostic test [55]. In 105 NSCLC patients, high tumor expression of miR-125a was strongly associated with worse overall and relapse-free survival, particularly in patients with p53-mutated tumors. Tumor miR-125a was inversely associated with IGFBP-3 mRNA and, although no survival data were presented for IGFBP-3, its low tumor expression was correlated with increased tissue Akt activation [55]. These examples illustrate strong published studies that point to the prognostic utility of measuring ncRNAs and their targets, but a large number of cases remain where the proposed prognostic benefit requires further substantiation.

### Therapy

Despite strong preclinical, and some clinical, evidence for the involvement of ncRNAs in many cancers, as well as other diseases, the development of ncRNA-based therapeutics is still in its infancy [106]. In an early first-in-human study, MRX34, a liposomal mimic of the putative tumor-suppressor, miR-34a, was investigated in 85 patients with various advanced solid tumors [107]. MiR-34a has been discussed earlier as being downregulated and sponged by HOTAIR, potentially in association with increased expression and secretion of oncogenic IGFBP-2 [48–50]. The MRX34 Phase 1 trial showed an overall response rate of 4% but a high level of immune-related adverse events led to cessation of the trial. Nevertheless a dose-related decrease in the combined expression of five miR-34a target genes was observed, leading the authors to conclude that the study demonstrated proof-of-principle for miRNA-based therapy [107]. MiR-155, an oncogenic miRNA that downregulates FOXO3a and IGFBP-1 [38], has been targeted in a Phase 1 trial of 66 lymphoma and leukemia patients using an oligonucleotide inhibitor MRG-106 (ClinicalTrials.gov NCT02580552). MRG-106 inhibits pathways downstream of IGF1R activation (ERK1/2, AKT, STAT3) but its role in these patients is unknown as no results have been posted. Many other potential ncRNA targets have been proposed for clinical trial but the future utility of this therapeutic approach in cancer remains to be established [108, 109].

## Concluding comments

In a rapidly-moving research area such as noncoding RNA biology, new discoveries occur frequently. Integrating these discoveries into an expanding framework that is meaningful biologically and can be exploited clinically is a growing challenge. In focusing on IGF and IGFBP biology as a target for ncRNA actions, this review has aimed to clarify a research area that may be developed to clinical advantage. As noted earlier, the vast majority of interactions defined so far have been restricted to cell biology studies, some with preclinical confirmation and limited clinical correlation in small patient cohorts.

While these discoveries have intrinsic value for the new biological insights they provide, the greatest benefit may come from those that can be confirmed in a clinical setting and refined to provide either a new tool for cancer prognosis, or a target for future drug development. Given the relatively disappointing outcomes from clinical trials directed towards modulating IGF1R signaling, there is reason to hope that an alternative approach to regulating this network of oncogenic pathways may come from regulating the ligands and their regulatory binding proteins, some of which also act through mechanisms independent of IGF1R signaling. This review has integrated many examples from within cancer biology, as well as some from non-cancer areas, to provide a framework that may be further exploited as a basis for future clinical utility to the benefit of cancer patients.
